# Ecological aspects of *Pintomyia fischeri* and *Migonemyia migonei* in municipalities with Canine Visceral Leishmaniasis, State of São Paulo, Brazil

**DOI:** 10.1590/S1984-29612023040

**Published:** 2023-07-17

**Authors:** Margareth Regina Dibo, Regiane Maria Tironi de Menezes, Fabiana Fernandes de Souza, Helio Benites Gil, Adriano Pinter

**Affiliations:** 1 Área Técnica de Doenças Vinculadas a Vetores e Hospedeiros Intermediários, Instituto Pasteur, São Paulo, SP, Brasil; 2 Divisão de Programas Especiais, Superintendência de Controle de Endemias, São Paulo, SP, Brasil; 3 Programa de Pós-graduação em Infectologia, Escola Paulista de Medicina, Universidade Federal de São Paulo – UNIFESP, São Paulo, SP, Brasil

**Keywords:** Sandfly, composition of species, seasonal activity, landscap scenarios, CVL, Flebotomíneos, composição de espécies, atividade sazonal, cenários da paisagem, CVL

## Abstract

The objective was to study the composition of sand fly species, the seasonal activity of the dominant species and correlation with average rainfall, sex association of the dominant species with domicile habitats and analyze different landscape scenarios for the two study sites in the municipalities of Itapevi and Mogi of the Crosses. Sandflies were captured with CDC(s) traps installed in three households at each study site for a period of 24 hours, biweekly for two years. A total of 2970 specimens were collected. The dominant species were *P.fischeri* and *M.migonei*. A statistically significant difference was registered between males and females of the two species in relation to the intra and peridomicile. The seasonal activity of both species showed a weak significant positive correlation with rainfall. The species *P.fischeri* and *M.migonei* may be potential vectors of CVL in the studied sites.

## Resumo

O objetivo foi estudar a composição das espécies de flebotomíneos, a atividade sazonal das espécies dominantes e a correlação com médias pluviométricas, associação de sexo das espécies dominantes com habitats domiciliares. Também analisar diferentes cenários da paisagem para os dois sítios de estudo nos municípios de Itapevi e Mogi das Cruzes. Os flebotomíneos foram capturados com armadilhas CDC(s) instaladas em três domicílios de cada sítio de estudo por um período de 24 horas, quinzenalmente por dois anos. Um total de 2.970 espécimes foram coletados. As espécies dominantes foram *P.fischeri* e *M.migonei*. Foi registrada diferença estatística significante entre machos e fêmeas das duas espécies em relação ao intra e peridomicílio. A atividade sazonal das duas espécies apresentou fraca correlação positiva significante com a chuva. As espécies *P.fischeri* e *M.migonei* podem ser potenciais vetores de LVC nos locais de estudo.

Visceral leishmaniasis is a zoonosis caused by the protozoan *Leishmania (Leishmania) infantum chagasi*, in the Americas, and is transmitted by the bite of infected sandflies (Diptera: Psychodidae: Phlebotominae). In studies carried out with strains of *Leishmania chagasi* from South America and *Leishmania infantum* from the Mediterranean, it was verified that both present the same DNA sequence, and it was proposed that *L (L.) chagasi* should not be considered a valid species; however, Laison and Shaw, based on its ecological characteristics, defended the maintenance of the parasitological entity at the subspecific level as *Leishmania (L.) infantum chagasi*. In Brazil, the disease initially had a rural character, but it has changed its epidemiological pattern in recent decades, with its introduction and expansion in urban environments. The main vector species is *Lutzomyia longipalpis*, but *Lutzomyia cruzi* has also been implicated in transmission, with the domestic dog (*Canis lupus familiaris*) being the main reservoir of this protozoan in urban settings (Lainson & Shaw, 2005; Lainson, 2010; Salomón et al., 2015).

In the state of São Paulo, the vector *L. longipalpis* was identified for the first time in 1997, in an urban area of the city of Araçatuba; the first case of canine visceral Leishmaniasis (CVL) was recorded in 1998 and, in 1999, the first autochthonous cases of human visceral Leishmaniasis (HVL) were diagnosed in this municipality (Camargo-Neves et al., 2001). Thereafter, the dispersal of the vector, of CVL and of HVL was reported from the northwest to southeast direction, in this state (Oliveira et al., 2016).

However, in the Metropolitan Region of São Paulo (RMSP), state of São Paulo, CVL occurs in rural areas close to forest fragments, which provides access for dogs to these fragments, in addition to socializing with other animals around the house, such as cats, swine, equine and chickens. In the state of Santa Catarina, Pinto et al. (2022) found the same situation in relation to dogs with CVL, that is, the presence and possible and possible entry into forest areas, in addition to the occurrence of poultry and swine farms in the vicinity of the houses. In the RMSP, sand fly collections were carried out in places where cases of CVL occurred, however, the *L. longipalpis* vector was only detected in the municipality of Caieiras (Pimont et al., 2015).

In this study, we aimed to know the composition and diversity of the sandfly species, the activity of dominant species during months of collection, the correlation of rainfall with the numerical abundance of dominant species, the association of the sex of these species with the surveyed households, and to compare landscape analyzes and potential influence on the sandfly species composition, of areas of two municipalities in the RMSP, with local transmission of CVL.

The study area comprises two locations in two municipalities of the RMSP, Itapevi, located west of the city of São Paulo, and Mogi das Cruzes, located east of the same city. In the municipality of Mogi das Cruzes, the first positive dog was identified in 2006, but other cases occurred in 2007 and 2008. In 2017, new cases of canine visceral leishmaniasis were detected in the rural area of Mogi das Cruzes in the neighborhoods Itapeti Residential Park. In that same year, cases of CVL began in dogs residing in the municipality of Itapevi in the neighborhoods Della Vitória Park, in some houses in the rural area that communicated with the same forest fragment.

**Figure 1:**
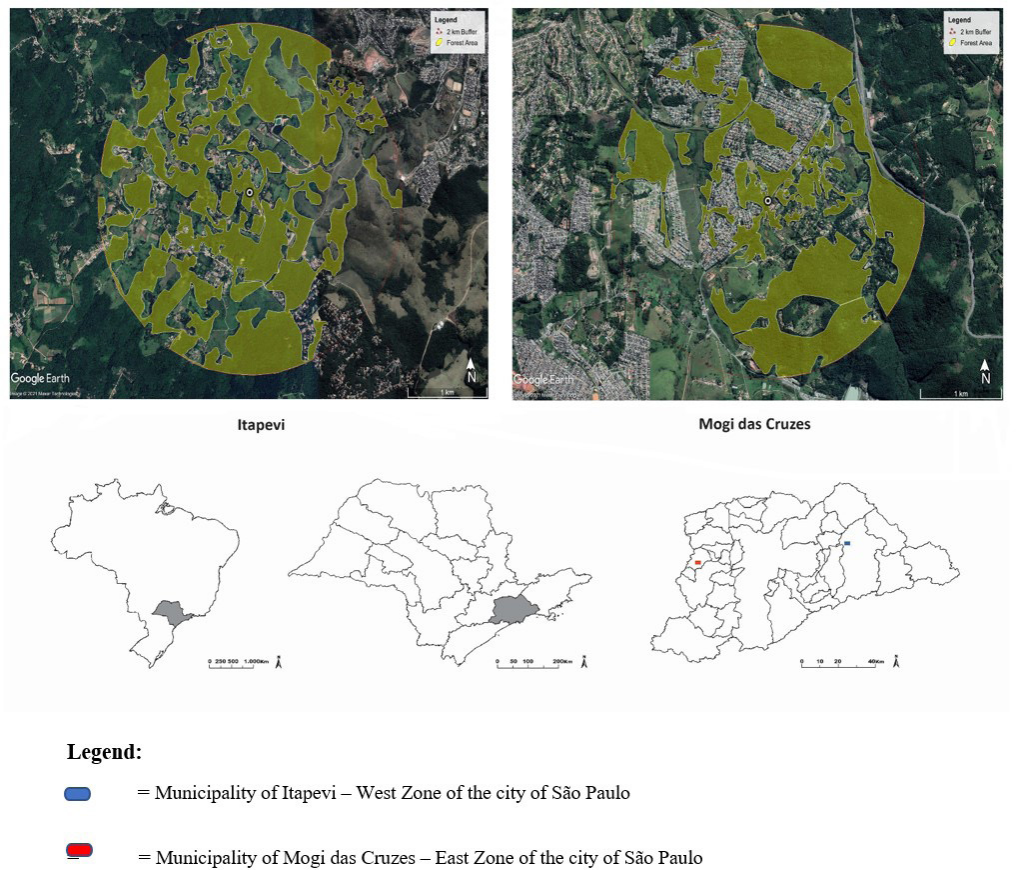
Map of the studied area, with landscape data analysis of the collect sites in Itapevi (left side) and Mogi das Cruzes (right side), MRSP, SP, Brazil.

Collections were carried out fortnightly in households in the two locations of the municipalities of Mogi das Cruzes and Itapevi. Two CDC(s) traps were installed for a period of 24 hours, every two weeks from September of 2017 to July of 2019, in intradomicile and peridomicile habitats, in three households, in each of the at each of the study sites. In the peridomicile, preferably, they were installed close to the place where a food source (dog, chicken) spent the night (Figure 1).

The Sandflys specimens collected with CDC(s) traps were transported from the field to the laboratory in trap cups packed in transport boxes. In the laboratory, the process of clarification, assembly and identification down to the species category was in accordance with Shimabukuro et al. (2011). The abbreviations for the genus and species were made in accordance with Marcondes (2007). The identified specimens were confirmed and deposited in the collection of the Department of Epidemiology of the FSP/USP. Data were recorded in an appropriate bulletin and entered into Microsoft Excel spreadsheets.

Diversity analysis was undertaken for composition of the phlebotomine species collected study sites, Margalef’s index (Mg), Simpson’s dominance index (Ds) and Shannon’s diversity index (H) were used for the measurements. Student's t test was used to calculate the variance and degrees of freedom, the latter always tending to infinity when dealing with Shannon's diversity (Magurran, 1988).

Williams’ average ( $\bar{x}$ w) (Forattini, 2002) was calculated for each month, for those species which occurred in greater abundance, taking into consideration the number of collections undertaken during each month for both study sites.

Pearson’s correlation (r) was used to verify the association between the monthly averages ( $\bar{x}$ w) of *P.fischeri* and *M.migonei*, with the accumulated rainfall of the month prior to the collections.

Chi-squared analysis, at the 5% significance level (α=0.05), was used to establish the existence or not of any association between the variable categories sex (male and female) and habitats (peridomicile and intradomicile) for dominant sandflies species.

The data were analyzed on Excel spread sheets. Statistical inferences were performed in softwares BioEstat (version 5.3).

In both two selected sites, the forested patches landscape parameters were identified by visual analysis of high-resolution satellite images (CNES/Astrium, DigitalGlobe and Terrametrics compositions, with passage date until October 2021), provided in Google Earth Pro 7.1.5.1557 (Google, Inc. Mountain View, CA, USA), polygons of the forest areas were scanned on screen, and the edge linear and total area calculations were performed in the same software. It was included in the metrics, the total area, in hectares, of all connected forest surrounding the studied site within a radius of 2 km, and the sum in kilometers of the total forest edge perimeters. All forest patches with no structural connection in between, but within a maximum distance of 100 meters from each other were included in the metrics.

During the study period, a total of 2,970 specimens were collected. In relation to the general totals, 1,928 (64.9%) were *P.fischeri* and 607 (20.4%) *M.migonei*. The third numerically dominant species is *P.lloydi*, with 259 (8.7%) specimens (Table 1) (Marcondes, 2007).

**Table 1:** Composition of sandfly species, by sex and habitat, collected in the in two locations, what are the neighborhoods Della Vitória Park and Itapeti Residential Park, respectively located in the municipalities of Itapevi and Mogi das Cruzes from 2017 to 2019.

	Della Vitória Park				Itapeti Residential Park					
Species	intra		peri		intra		peri			
	♀	♂	♀	♂	♀	♂	♀	♂	Total	%
*Brumptomyia* sp.	0	0	7	8	0	0	0	0	15	0.5
*B. nitzulescui*	9	4	4	1	3	1	0	1	23	0.8
*E.firmatoi*	0	5	0	0	0	0	3	1	9	0.3
M.* alphabetica	0	0	2	0	0	0	0	0	2	0.1
*M. migonei*	106	101	73	92	64	54	52	65	607	20.4
*N. intermedia*	2	0	2	3	3	2	5	0	17	0.6
*N. neivai*	33	26	21	9	0	0	0	0	89	3.0
*N. withmani*	1	0	0	1	0	0	0	1	3	0.1
P.* pascalei	0	0	1	0	0	0	0	1	2	0.1
P.** fischeri	340	241	224	266	148	44	554	111	1928	64.9
*P.pessoai*	3	0	0	0	0	0	0	0	3	0.1
*P. arthuri*	0	1	0	0	0	0	1	0	2	0.1
*P. lloydi*	115	77	23	37	0	2	3	2	259	8.7
*Psychodopygus* sp.	4	0	2	0	2	0	2	0	10	0.3
*P. geniculatus*	0	0	1	0	0	0	0	0	1	0.0
Total	613	455	360	417	220	103	620	182	2970	100.0
Richness (S) = N^o^ species or groups			15				10			
Margalef’s index (Mg)			1.86				1.28			
Shannon’s diversity index (H)			1.23				0.69			
Simpson’s dominance index (D)			0.40				0.62			

**Subtitle** – Abbreviation of Genres: *B.* = *Brumptomyia*, *E.* = *Expapillata*, M.* = *Martinsmyia*, *M.* = *Migonemyia*, *N.* = *Nyssomyia*, P.* = *Psathyromyia*; P.** = *Pintomyia*, *P.* = *Psychodopygus*.

The greatest species richness (S=15) and diversity were registered respectively by Margalef’s index (Mg=1.86) and Shannon’s index (H=1.23) for Della Vitoria Park in Itapevi. The highest Simpson's dominance (Ds=0.62) was estimated for the species collected in Itapeti Residential Park. (Table 1). Student's t-test indicated that the values of Shannon's diversity index (H) estimated for the species composition of sandflies, when comparing the localities of Della Vitoria Park (H=1.23) and Itapeti Residential Park (H=0.69), it was found to differ greatly and highly significant (t=8.2; p<0.001).

**Figure 2:**
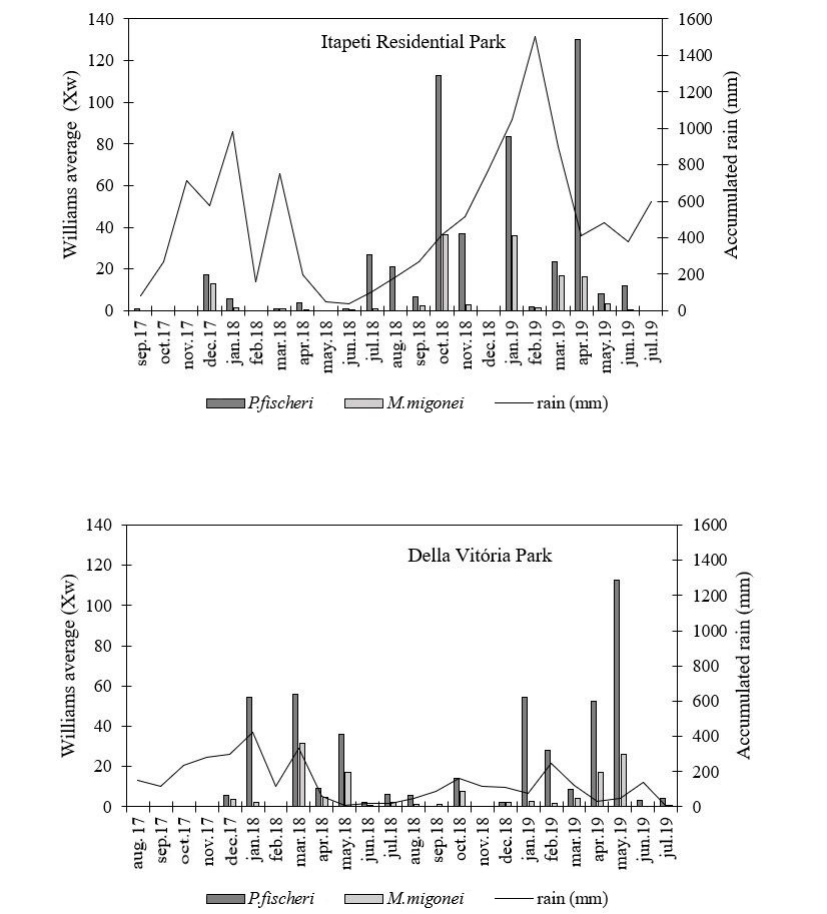
Variation of Williams mean of *P.fischeri* and *M.migonei* collected in different months, in two locations, what are the neighborhoods Della Vitória Park and Itapeti Residential Park, respectively located in the municipalities of Itapevi and Mogi das Cruzes from august 2017 to july 2019.

The highest peaks of activity recorded by Williams’ average (X ®_w_) measured over the months compared to rainfall in the month previous to each collection were found for the species *P.fischeri*, both in Della Vitoria Park and in Itapeti Residential Park. Spring and summer months were those of the most intense activity for both species (Figure 2).

Pearson's coefficient of correlation (r) presented a weak positive, though significant, correlation (p<0.05) for Williams' monthly average of *P.fischeri*, with the accumulated rainfall for the previous month on the occasion of each collection in Della Vitória Park (r = 0.45; p=0.03). Also, weak but significant was the correlation between the same variables for the specimens of *M. migonei* collected in Itapeti Residential Park (r = 0.43; p=0.04).

According to the categorical variable’s habitat and sex, it was observed that the species *P. fischeri* had a greater number of females than males, both in the intradomicile of Della Vitória Park and in the peridomicile of Itapeti Residential Park. For the *M.migonei* species, the number of females was greater and the males in the intradomicile of Della Vitória Park. The same is true for *Ps.lloydi* species (Table 1).

The chi-square distribution showed that there is a highly significant statistical difference between the female and male variables of the dominant species *P.fischeri* and *M.migonei*, in relation to the intra and peridomiciliary variables in Della Vitória Park and Itapeti Residential Park (χ2 = 285.8 , gl = 9, p <0.001).

The landscape metrics analysis yielded different scenarios for each site, the total remain forest area within 2 km radius and the total border forest perimeter were 595.64 ha and 95.00 km for Itapevi and 494.31 ha and 67.32 km for Mogi das Cruzes (Figure 1). In Itapevi, 47.4% of the area (within 2 km radium) is composed by remain forest patches whereas this value was 39.3% for Mogi das Cruzes, but in proportion, in Itapevi the total forest perimeter over total forest area was 0.16, in Mogi das Cruzes was 0.13. Therefore, Itapevi was characterized by occurrence of larger forest area and larger proportion of forest edge when compared to Mogi das Cruzes (Figure 1).

The most abundant species in Della Vitória Park and Itapeti Residential Park were also predominant in collections undertaken by other authors in in the MRSP. In Embu das Artes, a place with transmission of VLC, the majority of the specimens colleted corresponded to the species *P.fischeri*, however *M.migonei* was also present (Galvis-Ovallos et al., 2017). In São Paulo, *P.fischeri* was collected in the parks of the Cantareira, Alfredo Volpi and the Zoological Park, and *M.migonei* in the Zoological Park and of Anhanguera and Cantareira (Oliveira Castelo et al., 2015). The species *M. migonei* is permissive to several species of *Leishmania* spp. and Guimarães et al. (2016) have demonstrated in the laboratory that *M. migonei* is susceptible to the development of *Leishmania infantum*. In a study carried out in Fortaleza (CE), *M.migonei* was considered a potential vector of VL, along with *L. longipalpis* (Silva et al., 2014).

The greatest richness and diversity for the sandfly species registered for the Della Vitória Park may be due to the increase in breeding sites caused by the loss of leaves of tree species during the dry period, which are characteristic of the forest fragments at the study site (Barreto & Catharino, 2015), besides the landscape metrics showed that Della Vitória Park site had 47.4% of area composed by remain forest and larger proportion of forest edges, larger forest areas may increase the diversity of the sandflies and the larger proportion of forest edge may increase the contact of between households and forest patches (Figure 1).

In Itapeti Residential Park, which presents greater diversification of agricultural activities, expansion pasture areas and the proximity of households to urbanized areas reflect into smaller remain forest patches area, when compared to Della Vitória Park, which may explain there is less richness and diversity and the greatest dominance of species (São Paulo, 2013).

The significant value of the test of hypotheses (t=8.2; p<0.001) for Shannon’s diversity index, showed that in fact the diversity value for the phlebotomine species in Della Vitoria Park was greater than that estimated for Itapeti Residential Park. It is believed that this difference may be related to the different stages of the conservation of the forest fragments around the residences researched and a greater availability or variety of trophic niches in the peridomiciliary areas.

The recorded of the occurrence of a weak though significant correlation between the Williams’ averages of *P. fischeri* and *M. migonei* collected respectively in Della Vitória Park and Itapeti Residential Park, and the rainfall of the month prior to the collection, may be related to the humidity in the soil caused by the rains, an occurrence which probably aided the decomposition of the organic material there present thus creating appropriate places for the oviposition and favoring the development of immature forms, and the increase in the abundance of adult forms collected in the months subsequent to the rainiest ones, a condition which has already been observed by Almeida et al. (2010), for other species of sandflies.

The highest peaks of activity as measured by Williams’ averages registered for *P. fischeri* during the rainy periods, were preceded by accumulated rainfall values for the month which did not exceed 300 mm in Della Vitória Park - Itapevi or 200 mm in Itapeti Residential Park - Mogi das Cruzes (Figure 2). Very probably, the low average values for the activity of the dominant species observed in Itapeti Residential Park, may be related to the high rainfall peaks which preceded the collection. Some authors also point out that sandflies are benefited by moderate rainfall and that very intense rainfall delays development, consequently there will be a low abundance of adult specimens (Dias et al., 2007; Macedo et al., 2008).

The numerical dominance of *P. fischeri* and *M. migonei* (Table 1) for the intradomestic and peridomestic habitats in the two study sites may be related to the existence of favorable habitat for oviposition, present in the domiciles or in the forest fragments close to the domiciles. It is also easy for females to obtain blood and thus develop their gonotrophic cycle, due to the presence of humans and the canine reservoirs that are very present in both study sites. Pasanisi (2020) carried out the first serological canine survey for the municipality of Itapevi and found that among 104 dogs from the peridomestic environment, 16 of them were diagnosed seropositive, totaling 15.3% of the samples. The seropositive animals were submitted to parasitological tests to confirm Leishmania infection. The author mentions that in the entomological investigation carried out, no specimen of *L. longipalpis* was found, however the two species recorded here by us as numerically dominant were found.

Although *P. fischeri* and *M. migonei* use the blood of dogs and birds, they present a high degree of anthropophily (Gomes & Galati, 1989). In Parque Della Vitória in Itapevi, the abundance of the species *P. fischeri*, later associated with studies of competence and vectorial capacity, could explain the occurrence of the first autochthonous human case of visceral leishmaniasis in 2018, without the occurrence of the *L. longipalpis* (São Paulo, 2023).

Also in the case of Mogi das Cruzes, the great abundance of *P. fischeri* females in the peridomicile of Parque Residencial Itapeti (Table 1) may be related to the great attractiveness that these species have for performing the blood meal in dogs, since this domestic animal also was very frequent in the peridomicile habitats of this locality.

Galvis-Ovallos et al. (2017) carried out a vectorial capacity study in specimens of *P. fischeri* and *M. migonei* collected in Embu das Artes; concluded that the great attractiveness of dogs for *P. fischeri*, their susceptibility to infection by *Leishmania infantum*, life expectancy and predominance in the RMSP, made it clear that *P. fischeri* is a potential vector of this parasite in the region. In the same municipality, they sequentially collected sandflies and found infection by *L. infantum* PCR positive in *P. fischeri* (Galvis-Ovallos et al., 2021).

However, in an area of transmission of visceral leishmaniasis, in São Vicente Férrer, State of Pernambuco, females of the species *M. migonei* were found naturally infected by *L. infantum*, indicating that this species of sand fly can be the vector of visceral leishmaniasis in areas where the presence of *Lutzomyia longipalpis* was not detected (Carvalho et al., 2010). The three studies demonstrated the possibility of infection of *P. fischeri* and *M. migonei* by *L. infantum*, in addition to the attractiveness of *P. fischeri* for dogs, which is an indication of the importance of the numerical dominance of these two species of sandflies in an area of transmission of canine visceral leishmaniasis, as has already been recorded for the capture sites here in this study.

What we focus on in this study is limited, for future studies it should be increased the samples in relation to the number of households and also other habitats should be accessed, therefore it could validate the results for the municipalities of Mogi das Cruzes and Itapevi, here represented by neighborhood localities.

Certainly, a canine survey and an entomological survey in a larger number of households, with the collection of sandflies in other sites, in addition to those surveyed here, in which there was a confirmed of dogs infected with visceral Leishmaniasis; would give us a greater epidemiological view of the situation of disease in the studied cities.

Besides the numerical dominance, for *P. fischeri* and *M. migonei* and their respective seasonal activities, and frequencies in the home environment, we emphasize that for these species or others recorded here, in the studied sites, to be considered potential vectors of *Leishmania infantum chagasi*, in the reported conditions, it is necessary to isolate the parasite from collected sandflies specimens. Future studies must still be developed with the aim of verifying whether the aforementioned sandfly species are vectors based on parameters of competence and vectorial capacity.
